# Tibiofemoral articulation and axial tibial rotation of the knee after a cruciate retaining total knee arthroplasty

**DOI:** 10.1186/s43019-024-00224-7

**Published:** 2024-05-24

**Authors:** Guoan Li, Chaochao Zhou, Sophia Li, Jia Yu, Timothy Foster, Hany Bedair

**Affiliations:** 1 Orthopaedic Bioengineering Research Center, Newton-Wellesley Hospital/Massachusetts General Brigham, 159 Wells Avenue, Newton, MA 02459 USA; 2 Department of Orthopaedics, Massachusetts General Hospital, Boston, MA USA

**Keywords:** Total knee arthroplasty, Knee, Articular contact, Internal tibial rotation, Kinematics

## Abstract

**Purpose:**

Numerous research has reported that total knee arthroplasty (TKA) cannot reproduce axial tibial rotations of normal knees. The objective of this study was to measure the tibiofemoral articular contact motions and axial tibial rotations of TKA knees to investigate the mechanism causing the knee kinematics change of after TKAs.

**Methods:**

Eleven patients with unilateral cruciate retaining (CR) TKA were tested for measurements of knee motion during a weight-bearing flexion from 0° to 105° using an imaging technique. The tibiofemoral contact kinematics were determined using the contact points on medial and lateral surfaces of the tibia and femoral condyles. Axial tibial rotations were calculated using the differences between the medial and lateral articulation distances on the femoral condyles and tibial surfaces at each flexion interval of 15°.

**Results:**

On femoral condyles, articular contact distances are consistently longer on the medial than on the lateral sides (*p* < 0.05) up to 60° of flexion, corresponding to internal tibial rotations (e.g., 1.3° ± 1.0° at 15–30° interval). On tibial surfaces, the articular contact point on the medial side moved more posteriorly than on the lateral side at low flexion angles, corresponding to external tibial rotations (e.g., −1.4° ± 1.8° at 15–30° interval); and more anteriorly than on the lateral sides at mid-range flexion, corresponding to internal tibial rotations (e.g., 0.8° ± 1.7° at 45–60° interval). At higher flexion, articular motions on both femoral condyles and tibial surfaces caused minimal changes in tibial rotations.

**Conclusions:**

These results indicate that the axial tibial rotations of these TKA knees were mainly attributed to asymmetric articulations on the medial and lateral femoral condyles and tibial surfaces. The data can help understand the mechanisms causing axial tibial rotations of TKA knees and help improve implant designs for restoration of normal knee kinematics.

## Introduction

Restoration of normal knee kinematics is a major objective of contemporary total knee arthroplasty (TKA) for patients. Numerous patient follow-up studies have reported excellent clinical outcomes of the surgery. However, there are also studies that reported over 20% of patients were unsatisfied with the surgery [1, 2]. Mid-range flexion instability and limited range of motion are often referenced as functional complications in clinical studies [3–5]. Although various patient factors and surgical parameters have been extensively investigated, changes in knee kinematics after the surgery, such as the axial tibial rotations, are always measured to evaluate postoperative clinical outcomes of patients after TKAs.

Most kinematics studies of the knee after TKA surgeries have measured posterior femoral condyle translations during various functional activities of the knee [6–10]. Morphologically, the differences in translations of the medial and lateral femoral condyles projected on a tibial plane are used to calculate the axial tibial rotations [11, 12]. The “medial-pivoting” axial tibial rotation, corresponding to longer posterior translations of the lateral femoral condyle than the medial condyle, has been described as a characteristic motion pattern of the knee [13–17]. These kinematics studies demonstrated that it is a challenge for a TKA knee to restore normal knee kinematics [18]. For example, the TKA knees were shown to have limited axial tibial rotations during flexion compared with normal knees, regardless of the types of the implanted TKA systems [19, 20]. The changes of femoral condyle translations are often referred to as causing reduced axial tibial rotations. Recently, the mechanisms causing axial tibial rotations of normal knees during a weightbearing flexion were investigated by analyzing the tibiofemoral articulations of the knee [21, 22]. The data revealed that the axial tibial rotations of the knee were mainly attributed to the asymmetric articulations on the medial and lateral femoral condyles, especially at low flexion angles. This data could provide new insights into the investigation of axial tibial rotations of TKA knees.

However, few studies have investigated the physiological articulations, and no data has been reported on the association between the tibiofemoral articulations and axial tibial rotations of the TKA knees [23]. Therefore, the objective of this study was to investigate the in vivo articular contact motions on the femoral condyle and tibial surfaces, and calculate the axial tibial rotations using the tibiofemoral articulation data of TKA knees of patients recruited in our previous studies [18, 24]. It was hypothesized that the articulation patterns on the medial and lateral femoral condyles and tibial surfaces of TKA knees are associated to the post-operative axial tibial rotations of the knee.

## Methods

A total of 11 patients (7 males, 4 females) with advanced medial knee osteoarthritis (OA) who are scheduled for TKA surgery (6 left, 5 right) were included in our previous study [18, 24, 25]. The study was approved by our institutional review board and each patient provided written informed consent prior to participation. At the time of surgery, the patients’ average age was 61 ± 4 years (range: 51–73 years), average height was 173.5 ± 9.9 cm (range: 154.9–185.4 cm), and average body weight was 94.4 ± 14.5 kg (range 70.3–120.2 kg). Exclusion criteria included posttraumatic arthritis, rheumatoid arthritis, or valgus knees.

All the OA knees received a cruciate retaining (CR) TKA (NexGen CR-Flex, Zimmer, Warsaw, IN) using a conventional surgical procedure [18, 24, 25]. The intraoperative extension-flexion gaps of each knee were well balanced at 0° and 90° of flexion using a mechanical alignment, and no posterior cruciate ligament (PCL) resection was performed. All patients returned for measurements of knee motions during a weight-bearing single leg lunge using a previously validated dual fluoroscopic imaging system (DFIS) at 8 ± 2.5 months after the surgery (range: 7–15 months). No patient had experienced any surgical complication, dislocation, or component subluxation.

**Fig. 1:**
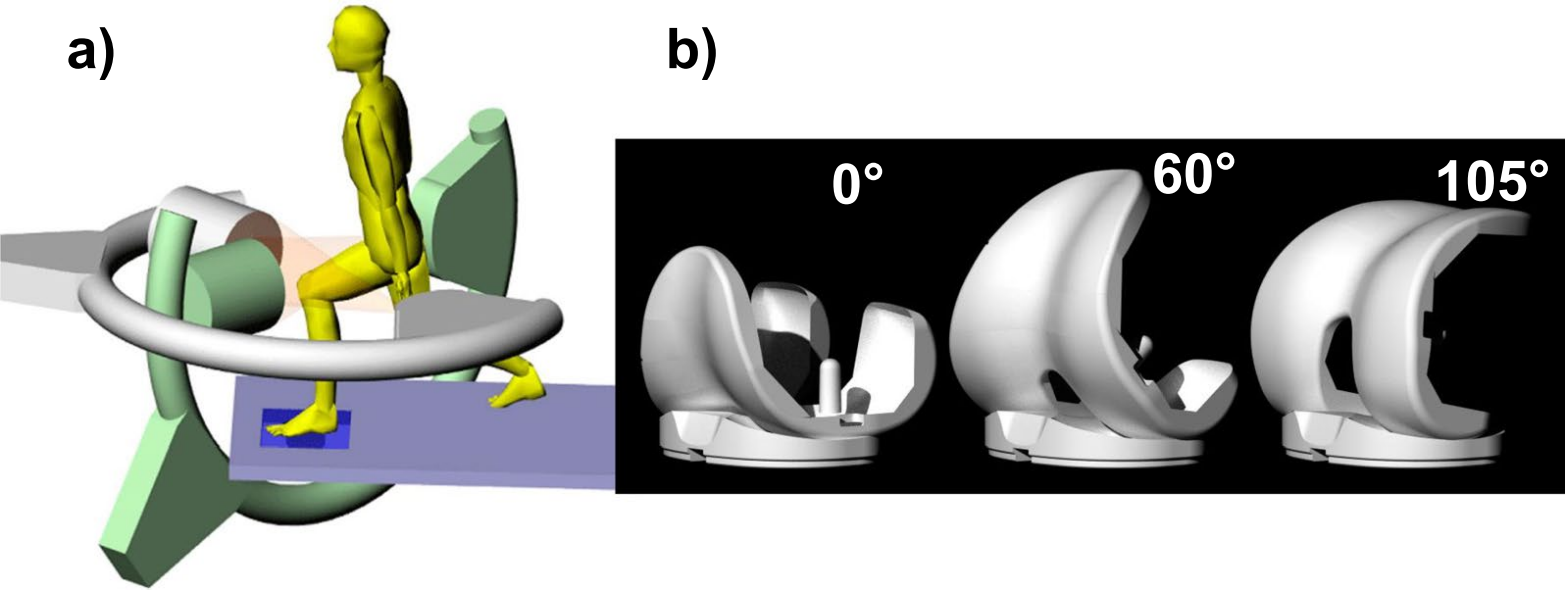
**a** The dual fluoroscopic imaging system (DFIS) set-up for measurements of patient knee joint positions during weight-bearing flexion. **b** In vivo TKA positions at different flexion angles along the flexion path reproduced using 3D CAD models of the TKA component

During the kinematics measurements, each subject was instructed to perform a weight-bearing, quasi-static single-leg lunge from full extension (0°) to maximal flexion (105°) at every 15° increments (Fig. 1a). The subject was instructed to maintain the orientations of the foot and torso during the experiment. The subject flexed the knee to a target flexion angle and held for 1 s for DFIS imaging and then flexed to another position. Following the experiment, the series of paired fluoroscopic images were imported into a solid-modeling software to establish a virtual fluoroscopic setup [26]. The 3D CAD models of the TKA component were also imported and individually manipulated in six degrees of freedom (6-DOF) until their projections matched the contours of the component on the dual fluoroscopic images captured during the actual weight-bearing flexion [27]. The process was repeated for all target flexion angles and at maximum flexion. The in vivo knee joint positions along the flexion path were then reproduced by a series of 3D CAD models of the TKA component (Fig. 1b). This technique was previously validated with an error of < 0.1 mm in measurements of joint translations and < 0.3° of joint rotations [26].

**Fig. 2:**
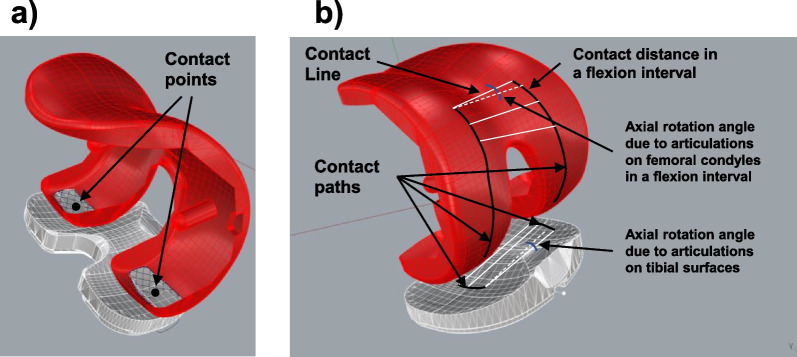
**a** Articulation contacts points on the medial and lateral femoral condyles and tibial surfaces of the TKA component at a flexion position. **b** Articular contact paths on the medial and lateral femoral condyles and tibial surfaces of the TKA component during flexion of the knee. Contact lines were illustrated at different flexion positions. The axial tibial rotation due to articulations on femoral condyles or tibial surfaces at each flexion interval was calculated as the angle between the two adjacent contact lines on the femoral condyles or tibial surfaces of the TKA component

At each target flexion position, the tibiofemoral joint contact area was determined as the area of the penetration of the femoral component into the tibial liner surface as described in our previous investigation (Fig. 2a) [28]. Articular contact points were defined as the centroids of the contact areas on the tibial and femoral component surfaces. A contact line on the femoral condyles or tibial surfaces was defined at each targeted flexion angle by connecting the medial and lateral contact points (Fig. 2b). The contact points moved on both femoral condyles and tibial surfaces during knee flexion. The medial or lateral articular contact motions were measured using the distances travelled by the contact points in each flexion interval of 15° on the femoral condyles and tibial surfaces [21, 22] (Fig. 2b). The contact points on both the femoral condyles and tibial surfaces represent a physiological description of articular contact motions of the knee.

A joint coordinate system was established for each TKA knee [27, 29]. The tibial long axis was parallel to the long stem of the tibial component. The tibial plane was defined as the plane perpendicular to the long axis. The medial–lateral axis was defined as a line connecting the centroids of the medial and lateral tibial surfaces. The anterior–posterior axis was perpendicular to the two axes in the tibial plane. The femoral long axis was defined along the long axis of the femoral component. The medial–lateral axis of the femoral component was defined as the flexion axis. The flexion angle was measured between the tibial and femoral long axes in sagittal plane.

Like our previous investigations of normal knees, the axial tibial rotation of the TKA knee with flexion was composed of two parts, one due to articulations on the femoral condyles and one due to articulations on the tibial surfaces (Fig. 2b). The axial tibial rotation angle about the tibial long axis, caused by articulations on the femoral condyles, was first calculated at each flexion interval as the angle between the contact lines at the beginning and the end of the flexion interval on the femoral condyles (Fig. 2b), where a longer articulation on the medial condyle corresponds to an internal tibial rotation. The axial tibial rotation angle due to articulations on the tibial surfaces was calculated using the contact lines at the beginning and the end of the flexion interval on the tibial surfaces (Fig. 2b), where a longer posterior contact distance on lateral side corresponds to an internal tibial rotation. The axial tibial rotations that resulted from articulations on the femoral condyles and tibial surfaces were calculated separately at each flexion interval and then summed to represent the total axial tibial rotation in the flexion interval. Finally, the axial tibial rotations at all flexion intervals were added up to calculate the overall axial tibial rotations along the knee flexion path.

Descriptive statistics were presented in the form of mean values and standard deviations (SDs). The articular contact distances on the medial and lateral sides were compared for both the femoral condyles and tibial surfaces. The axial tibial rotations due to articulations on the femoral condyles and tibial surfaces were specifically compared at each flexion interval of 15° and along the flexion path from 0° to 105° of flexion. A repeated measure analysis of variance (ANOVA) was used for data analysis. Significance level was set at *α* = 0.05. The statistical analyses were conducted using SPSS software (SPSS Inc., Chicago, Illinois, USA).

## Results

**Fig. 3:**
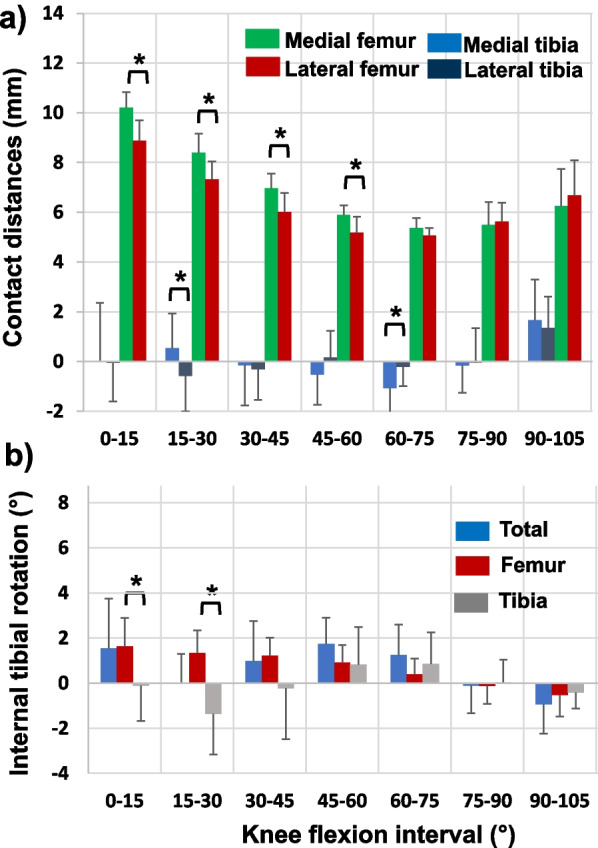
**a** Articular contact distances (mean ± SD) on the medial and lateral femoral condyles and tibial surfaces of the TKA component. A “*” indicates that the contact distance is significantly different between the medial and lateral femoral condyles or tibial surfaces (*p* < 0.05). **b** Internal (+) and external (−) tibial rotations (mean ± SD) at all flexion intervals of the TKA knee. A “*” indicates that the internal tibial rotation due to articulations on the femoral condyles is significantly different from that due to articulations on tibial surfaces of the TKA component (*p* < 0.05)

Articulation distances on both medial and lateral femoral condyles decreased in each flexion interval with flexion until 60° (Fig. 3a). On average, the articulations on the medial femoral condyle were longer than those (but with magnitudes < 1.3 mm) on the lateral side at each flexion interval (*p* < 0.05) (Table 1). At higher flexion angles, the contact distances similarly increased with flexion on both medial and lateral condyles (*p* > 0.05). The articulations on tibial surfaces were smaller than those on the femoral condyles at each flexion interval along the flexion path (*p* < 0.05) (Fig. 3a). On average, the articulations on tibial surfaces were less than 1.1 mm in magnitude at each flexion interval until 90°. For example, the contact points moved 0.5 ± 1.4 mm posteriorly on medial and −0.6 ± 1.4 mm anteriorly on lateral tibial surfaces from 15° to 30° (*p* < 0.05). From 60° to 75°, the contact points moved anteriorly −1.1 ± 1.0 mm on medial and −0.2 ± 0.8 mm on lateral tibial surfaces (*p* < 0.05). No significant difference in contact distances was found between the medial and lateral tibial surfaces in other flexion intervals (Table 1). Beyond 90°, the articulations in posterior direction on both medial and lateral tibial surfaces increased similarly (*p* > 0.05). On average, the magnitudes of differences of the contact distances between the medial and lateral sides are similar (< 1.3 mm) for the femoral condyles and the tibial surfaces except at 0–15° flexion interval (Table 1).

**Table 1:** Differences of contact distances between medial and lateral (med–lat) femoral condyles (mean, SD) and tibial surfaces (mean, SD) of the TKA component at different flexion intervals and the entire flexion path (0–105°)

			Flexion intervals					
	0–15°	15–30°	30–45°	45–60°	60–75°	75–90°	90–105°	0–105°
Med–lat (mm)								
Femur	1.32*	1.07*	0.96*	0.71*	0.30	− 0.12	− 0.42	3.22*
SD	1.03	0.82	0.63	0.58	0.54	−0.63	−0.76	4.00
Tibia	0.08	1.10*	0.17	−0.69	−0.86*	−0.12	0.32	0.02
SD	1.26	1.47	1.83	1.33	1.05	−0.76	−0.60	0.48

A “*” means a significant difference between the medial and lateral articular contact distances (*p*<0.05)

The total internal tibial rotation was 1.5 ± 2.2° at flexion interval of 0–15°, where 1.6° ± 1.3° was due to articulations on the femoral condyles and −0.1° ± 1.6° due to articulations on the tibial surfaces (Fig. 3b, Table 2). At flexion interval of 15–30°, the total internal tibial rotation was 0.0° ± 1.3°, where 1.34° ± 1.0° was due to articulations on the femoral condyles and −1.36° ± 1.8° on the tibial surfaces. The internal tibial rotation due to articulations on the femoral condyles is significantly different (*p* < 0.05) with that on the tibial surfaces at each flexion interval until 30° of flexion. At flexion interval of 60–75°, the total internal tibial rotation was 1.25 ± 1.3°, where 0.4 ± 0.7° was due to articulations on the femoral condyles and 0.85 ± 1.4° on the tibial surfaces (*p* > 0.05). At flexion intervals beyond 75° of flexion, minimal axial tibial rotations were measured by articulations on either femoral condyles or tibial surfaces at each flexion interval.

**Table 2:** Internal (+) and external (−) tibial rotations (mean ± SD) at difference flexion intervals and entire flexion path due to articulations on femoral condyles and tibial surfaces

			Flexion intervals					
	0–15°	15–30°	30–45°	45–60°	60–75°	75–90°	90–105°	0–105°
Internal tibial rotation (ITR) (°)								
Femur	1.65	1.34	1.21	0.92	0.40	−0.13	−0.52	4.86
SD	1.25	1.00	0.81	0.77	0.69	0.78	0.97	4.21
Tibia	−0.10	−1.36	−0.23	0.82	0.85	0.03	−0.41	−0.40
SD	1.58	1.81	2.26	1.67	1.41	1.01	0.71	4.42
*p*values	0.01	0.01	0.14	0.90	0.43	0.71	0.77	0.05

A *p*-value indicates a statistical difference of tibial rotations caused by articulations on femoral condyles and tibial surfaces

**Fig. 4:**
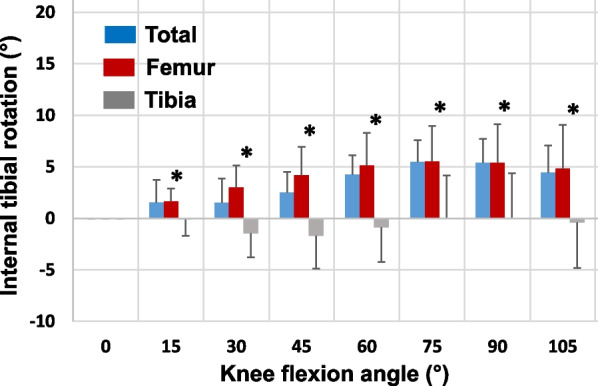
Overall internal tibial rotation (mean ± SD) along the flexion path of the TKA knee, and the rotation components measured using articular contact motions on the femoral condyles and tibial surfaces. A “*” indicates that the axial tibial rotation due to articulations on femoral condyles is significantly larger (*p* < 0.05) than that due to articulations on the tibial surfaces along the flexion path

The overall axial tibial rotations with knee flexion angles were shown to consistently increase with knee flexion (Fig. 4). At 30° of flexion, the overall internal tibial rotation was 1.5 ± 2.3° where 3.0 ± 2.1° was due to articulations on the femoral condyles and −1.7 ± 3.2° due to articulations on the tibial surfaces (*p* < 0.05). At 75° of flexion, the overall internal tibial rotation was 5.5 ± 2.1° where 5.5 ± 3.5° was due to articulations on the femoral condyles and 0.0 ± 4.2° due to articulations on the tibial surfaces (*p* < 0.05). At higher flexion angles, the overall axial tibial rotations slightly decreased and on average, the articulations on the tibial surfaces made little contribution to overall tibial rotations.

## Discussion

This study found that the asymmetric articular distances on the medial and lateral femoral condyles are associated with internal tibial rotations until mid-range flexion of the knee. The asymmetric articulations on the medial and lateral tibial surfaces were associated with external tibial rotation at early flexion and internal tibial rotation at mid-range flexion in this group of patients. The data proved the hypothesis that the articulation patterns on the medial and lateral femoral condyles and tibial surfaces after the TKA are associated with the postoperative axial tibial rotations of the knee.

Numerous investigations have reported on the axial rotations of the knee after TKAs during full range of knee flexion [6, 7, 9, 11, 30, 31]. A recent study extensively summarized the axial tibial rotation data of most existing TKA systems reported in literature [19]. These in vivo TKA kinematics studies indicated that in general, the range of axial tibial rotations of contemporary TKA knees is less than 7°. Our current study measured an axial tibial rotation of 5.5° during flexion of the TKA knees, that is consistent with the axial tibial rotation data of the TKAs reported in literature.

Most in vivo studies of normal human knee kinematics have reported that knee flexion is coupled with an internal tibial rotation in the range of 1–20° [21, 32, 33]. The ranges of internal tibial rotations of TKA knees are much smaller than those observed in normal human knees. Inability to restore normal knee kinematics in TKA knees has been widely recognized to affect knee function and clinical outcomes. Numerous innovations of TKA designs and surgical techniques have been proposed to improve knee kinematics. For instance, novel design concepts have been proposed to mimic the medial pivoting rotation feature of normal knees [13, 14, 34, 35]. Bi-cruciate retaining or substituting designs have been proposed to better maintain major passive structures of the knee [20, 36–40]. Recently, kinematic alignment (KA) technique for implantation of the TKA components has been advocated to enhance clinical outcomes [30, 41, 42]. However, patient’s follow-up studies have not shown dramatic improvement in axial tibial rotations compared with conventional TKA systems [19, 20]. These observations indicate that there could exist other intrinsic biomechanical features that determine axial rotations of the knee during flexion.

In literature, few previous research measured the tibiofemoral joint articular contact motions and reported the contact point positions on the tibial surfaces [12, 43–45]. However, tibiofemoral joint articulation includes contact motions on both tibial and femoral condyle surfaces. Our recent studies determined contact positions on both the medial and lateral femoral condyles and tibial surfaces of normal knees [21]. We found that the variations of contact positions were similar between the medial and lateral tibial surfaces during the knee flexion. We also found that the contact distances on the medial femoral condyles were significantly larger than those on the lateral femoral condyles. For example, at the flexion interval of 0–15°, the articular distance on the medial femoral condyle was ~ 4.1 mm longer than that on the lateral condyle. We further found that the differences in contact distances on the medial and lateral femoral condyles at low flexion angles correspond to increases in internal tibial rotations. The data on normal knee articular kinematics revealed that the axial tibial rotations are mainly associated with the asymmetric articulations on the medial and lateral femoral condyles. The articular contact on the tibial surfaces minimally affects axial tibial rotations compared with the articulations on the femoral condyles. Therefore, the articulation on femoral condyles is an important factor that could result in axial tibial rotations of normal knees.

The current study found that in TKA knees, the differences of contact distances on medial and lateral femoral condyles are much smaller compared with those observed in normal knees. For example, at the flexion interval of 0–15°, the contact distance in medial condyle is 1.32 mm longer than the lateral side in the TKA knees that is smaller than that (4.1 mm) of normal knees. Correspondingly, the axial tibial rotation of 1.65° of the TKA knees is also much smaller than that observed in normal knees (6.1°) [21]. Interestingly, the difference in contact distances between the medial and lateral femoral condyles of the TKA at flexion interval of 15–30° is similar in magnitude, but opposite in direction to that measured on tibial surface of the TKA knee. The articulation on the tibial surface caused external tibial rotation that is opposite but similar in magnitude to that caused by articulation on the femoral condyles. Therefore, the total axial tibial rotation was small in this flexion interval. At the flexion interval 60–75°, however, the articulations on the tibial surface caused similar internal tibial rotation with that caused by articulations on the femoral condyles. In most flexion intervals, the axial tibial rotations due to articulations on tibial surfaces are similar in magnitudes with those due to articulations on femoral condyles, as shown in Table 1. This kinematic feature is drastically different from that of normal knees where the articulations on tibial surfaces contributed negligibly to axial tibial rotations compared to that on femoral condyles [21]. The fact that the articulations on tibial surfaces caused opposite axial tibial rotations in intervals at low flexion angles such as 15–30° and in mid-range flexion such as 60–75° imply that the axial tibial rotations were unstable along the flexion path. Their contributions to overall axial tibial rotations are therefore minimal during the entire flexion of the TKA knees as shown in Fig. 4.

These discussions revealed an interesting biomechanical phenomenon of TKA knee kinematics. It is the reduced differences between articulations on the medial and lateral femoral condyles after the TKA compared to normal knees that correspond to reduced axial tibial rotations. Therefore, restoration of the tibiofemoral articulation patterns of normal knees after TKAs could be an important biomechanical goal that could help improve the axial tibial rotations of the TKA knees. Both geometric features of the tibiofemoral articular surfaces and surgical implantation techniques of the TKA components could play important roles affecting the articulation behavior of the knee. A quantitative evaluation of each biomechanical factor that may influence joint articulations is critical for improvement of the functional biomechanics of the TKA system. Future research is warranted on improvement of articulation features of contemporary TKA systems that is aimed to restore normal knee kinematics.

There are several limitations that need to be noted when interpreting the data of this paper. Only 11 patients operated on using one single CR TKA system were involved in this study. Future studies should include larger sample sizes, other popular types of TKA systems of different articulation designs, and implantation techniques (including mechanical alignment and kinematic alignment techniques) to verify the mechanisms that cause reduced axial tibial rotations in TKA knees. Future studies should also analyze the effects of gender, laterality, body height and body weight on the knee joint kinematics. This study only measured the knee kinematics during a single leg flexion at flexion intervals of 15°. Articulations of the knee during other functional activities, such as gait, stair climbing and descending, etc., should be investigated to examine the articular behavior of the knee under various in vivo loading conditions. The changes of articular contact kinematics should also be analyzed in smaller flexion intervals to examine the variations in articulation surfaces along the flexion path. Finally, the contact kinematics was measured using the position of the centroid point of the contact area. Therefore, comparison of contact kinematics between different studies must use data measured using the same measurement method. Despite these limitations, this study provides important insights into the investigation of intrinsic biomechanical mechanisms that could affect the functional kinematics behavior of the knee after TKAs.

## Conclusion

These results indicate that the axial tibial rotations of these TKA knees were mainly attributed to asymmetric articulations on the medial and lateral femoral condyles and tibial surfaces. The data can help understand the mechanisms causing axial tibial rotations of TKA knees and help improve implant designs for restoration of normal knee kinematics.

## Data Availability

Not applicable.
